# Association Between Retinal Layer Thickness and Cognitive Decline in Older Adults

**DOI:** 10.1001/jamaophthalmol.2022.1563

**Published:** 2022-05-26

**Authors:** Hyeong Min Kim, Ji Won Han, Young Joo Park, Jong Bin Bae, Se Joon Woo, Ki Woong Kim

**Affiliations:** 1 Department of Ophthalmology, Seoul National University College of Medicine, Seoul National University Bundang Hospital, Seongnam, Republic of Korea; 2 Department of Neuropsychiatry, Seoul National University Bundang Hospital, Seongnam, Republic of Korea; 3 Department of Ophthalmology, Kangwon National University School of Medicine, Kangwon National University Hospital, Chuncheon, Republic of Korea; 4 Department of Psychiatry and Behavioral Science, Seoul National University College of Medicine, Seoul, Republic of Korea; 5 Department of Brain and Cognitive Science, Seoul National University College of Natural Sciences, Seoul, Republic of Korea

## Abstract

**Importance:**

Retinal layer thickness is hypothesized to be related to cognitive function in patients with mild cognitive impairment (MCI) and Alzheimer disease (AD). However, longitudinal cohort studies of the healthy older population are scarce.

**Objective:**

To investigate the association between retinal layer thickness and cognitive impairment and future cognitive decline in a community-based population cohort.

**Design, Setting, and Participants:**

A total of 430 randomly sampled community-dwelling Korean individuals 60 years or older participated in the baseline assessment (mean [SD], 76.3 [6.6] years) 215 of whom completed a mean (SD) of 5.4 (0.6) years (range, 4.1-6.2 years) of follow-up. Using spectral-domain optical coherence tomography, the study team assessed the thickness of 6 retinal layers in the macular region, the peripapillary retinal nerve fiber layers (RNFLs), and the subfoveal choroid at baseline.

**Exposures:**

Age, sex, education, diabetes, hypertension, and apolipoprotein E4 gene status.

**Main Outcomes and Measures:**

Retinal layer thickness and cognitive function test scores were analyzed.

**Results:**

This study included 430 participants (female, 208 [48.6%]). Baseline macular RNFL thickness was associated with baseline Consortium to Establish a Registry for Alzheimer’s Disease (CERAD) score (coefficient [β] = 0.077; 95% CI, 0.054-0.100; *P* = .04 for total macular area) and Mini-Mental State Examination (MMSE) score (coefficient [β] = 0.082; 95% CI, 0.063-0.101; *P* = .03 for total macular area). A thinner baseline total macular RNFL thickness (lowest quartile, <231 μm) was associated with a larger decline in the CERAD and MMSE scores during the follow-up period (*P* = .003 and *P* = .01, respectively). Furthermore, participants with baseline total macular RNFL thickness below the lowest quartile cutoff value presented a greater decline in cognitive scores and a higher prevalence of cognitive impairment and Alzheimer disease than those with RNFL thickness above the lowest quartile cutoff value.

**Conclusions and Relevance:**

In this study, macular RNFL thickness could be used as a prognostic biomarker of long-term cognitive decline in adults 60 years or older. However, to confirm these results, further large-scale population-based studies should be performed.

## Key Points

**Question:**

Is retinal layer thickness associated with cognitive decline in an older population?

**Findings:**

In this cohort study including 430 community-dwelling participants in Korea, baseline macular retinal nerve fiber layer (RNFL) thickness was associated with baseline cognitive function scores and follow-up cognitive decline.

**Meaning:**

These findings suggest that macular RNFL thickness could be considered a predictive biomarker for evaluating cognitive function in older individuals.

## Teaser

This cohort study investigates the association between retinal layer thickness and cognitive impairment and future cognitive among 430 randomly sampled community-dwelling participants in Korea.

## Introduction

Alzheimer disease (AD) is a severe neurodegenerative disorder affecting millions of people globally.^1^ Subjective cognitive decline, also described as preclinical AD, is likely to progress over time to mild cognitive impairment (MCI) and AD.^2^ Therefore, clinicians and researchers have investigated several biomarkers of AD to diagnose the disease as early as possible. Certain structural brain magnetic resonance imaging findings,^3^ and cerebrospinal fluid–based biomarkers^4^ have been identified. The apolipoprotein E (*APOE ε4*) genotype has been recognized as a notable biomarker for AD. Moreover, many efforts have been made to discover noninvasive ophthalmic biomarkers for the identification of patients with cognitive impairment and AD.

Since Parisi et al^5^ observed peripapillary retinal nerve fiber layer (RNFL) thinning in patients with AD, other studies have described optical coherence tomography (OCT) parameters in patients with dementia, MCI, and preclinical AD. Most studies have reported peripapillary RNFLs thinning in patients who have AD and MCI compared with their age-matched controls.^6,7,8,9,10,11,12,13,14^ Recent advances in the visualization and segmentation of retinal layer structures by OCT have provided better opportunities to analyze retinal layer morphology in vivo.^15,16^ Prior investigations have reported correlations between the peripapillary RNFLs, ganglion cell (GC)–inner plexiform layer (IPL), and other retinal layers and cognitive impairment in patients with MCI or AD who showed retinal layer thinning.^5,6,7,8,9,10,11,12,13,14,17,18,19,20^ Population-based studies conducted by the UK Biobank suggested that thinner RNFLs could indicate baseline cognitive dysfunction and future cognitive decline over time, leading to an increased risk of AD development.^21^

OCT angiography was developed and the association between cognitive performance and changes in the retinal microvasculature was investigated. Yoon et al^22^ suggested that patients with AD had reduced macular vessel and perfusion density in the superficial capillary plexus compared with patients who had MCI and healthy controls. O’Bryhim et al^23^ proposed that cognitively healthy participants with preclinical AD presented with foveal avascular zone (FAZ) enlargement, and their most recently published 3-year longitudinal follow-up results showed that the FAZ enlargement remained, although no differences were found in the other retinal structural parameters.^24^

Similar to the UK Biobank studies, this community-based longitudinal cohort study conducted in Korea aimed to enroll a large number of participants to determine the association between retinal layer thickness and cognitive function in healthy individuals and individuals with cognitive impairment. Both cross-sectional and longitudinal follow-up studies were conducted to determine relevant retinal layer thickness parameters.

## Methods

The institutional review board of Seoul National University Bundang Hospital approved this population-based longitudinal cohort study, which adhered to the tenets of the Declaration of Helsinki. Written informed consent was obtained from all participants. For the cohort study, we used the Strengthening the Reporting of Observational Studies in Epidemiology (STROBE) reporting guideline. The study participants did not receive any compensation or incentives.

### Participants

We enrolled Korean adults 60 years and older from 2 population-based longitudinal cohort studies: the Korean Longitudinal Study on Health and Aging (KLoSHA)^25^ and the Korean Longitudinal Study on Cognitive Aging and Dementia (KLOSCAD).^26^ The specific details of these studies are described in eMethods 1 in the Supplement. A total of 500 participants were enrolled, and 70 were excluded because of high myopia (axial length >26 mm), high intraocular pressure (>21 mm Hg), self-reported glaucoma history, and combined ocular pathologies that might affect retinal layer thickness, such as age-related macular degeneration, epiretinal membrane, diabetic macular edema, and diabetic retinopathy found on OCT infrared imaging, leaving 430 participants for the final analysis. Among these 430 participants, 215 completed follow-up assessments, with a mean (SD) follow-up duration of 5.4 (0.6) years (range, 4.1-6.2 years) (eFigure 1 in the Supplement).

### Ophthalmic and Cognitive Function Assessments

We conducted a baseline assessment from September 2010 to September 2011 and a follow-up assessment from September 2015 to September 2016. The baseline assessment comprised comprehensive ophthalmic examinations, including spectral-domain OCT (SD-OCT) to determine the best-corrected visual acuity, intraocular pressure, auto kerato-refractometry, optical biometry axial length calculation (IOL Master; Carl-Zeiss Meditec), and SD-OCT (Spectralis; Heidelberg Engineering). The macula protocol, consisting of a raster scan composed of 31 horizontal lines centered on the fovea, was performed with 25 frames averaged for each OCT B-scan, along with 6-mm length and automatic real-time processing^27^ to obtain the retinal layer thickness of both the macula/fovea and peripapillary areas. Nine macular fields were adopted in the macula/fovea area based on the Early Treatment Diabetic Retinopathy Study group, consisting of 3 concentric rings centered on the fovea measuring 1 mm (center), 3 mm (inner), and 6 mm (outer) (eFigure 2 in the Supplement). We measured 6 individual retinal layers (RNFL-GC layer, IPL, inner nuclear layer, outer plexiform layer, and outer nuclear layer) using a built-in automated program (HEYEX software; eFigure 2 in the Supplement). In our study, the outer nuclear layer included the thick hyporeflective band, the anatomical outer nuclear layer, and the Henle fiber layer, in accord with the terminology before the 2014 International Nomenclature for Optical Coherence Tomography Panel consensus.^28^ Two independent retina specialists (H.M.K. and Y.J.P.) manually measured the subfoveal choroidal thickness using the enhanced-depth imaging mode, and the average of repeated measures was analyzed. We obtained the average thickness of the peripapillary RNFLs measured in 6 different areas (eFigure 2 in the Supplement). We also collected and analyzed retinal thickness data for the outer ring, inner ring, and total macular area. The right eye of each participant was selected for analysis of the retinal thickness data.

Research neuropsychologists or trained research nurses performed comprehensive neuropsychological assessments, including the Korean version of the Consortium to Establish a Registry for Alzheimer’s Disease Assessment Packet (CERAD-K) and the Mini-Mental State Examination (MMSE) (eMethods 2 in the Supplement). A panel of research neuropsychiatrists diagnosed dementia according to the diagnostic criteria of the *Diagnostic and Statistical Manual of Mental Disorders* (Fourth Edition).^29^ MCI was diagnosed according to the consensus criteria from the International Working Group on MCI.^30^ The presence of objective cognitive impairment was ascertained when the performance of the participants was −1.5 SD or below the age-, sex-, and education-adjusted norms in any of the neuropsychological tests. We classified participants into cognitively normal and cognitively impaired (MCI or dementia) groups.

### Statistical Analysis

Continuous variables were compared between the groups using *t* test and categorical variables were compared using Pearson χ^2^ test. Continuous variables within participants were compared using paired *t* tests. We examined the associations between baseline retinal layer thickness and baseline cognitive test scores (CERAD total score and MMSE) using multiple linear regression analyses and changes in cognitive test scores using repeated-measures analysis of variance. In addition, the lowest quartile of baseline macular RNFL thickness was set to compare the 2 groups below and above the cutoff value. In both analyses, we adjusted for age, sex, level of education, presence of the *APOE* ε4 allele, diabetes, and hypertension as covariates. Two-sided hypothesis testing was performed, and the null hypothesis was rejected if *P* = .05; the *P* values were not adjusted for multiple analyses. The 95% CI was also documented. All statistical analyses were performed using IBM SPSS Statistics for Windows (version 25.0; SPSS Inc).

## Results

This study included 430 participants (female, 206 [48.6%]). The demographic and clinical characteristics of the study participants are summarized in Table 1. Cognitive disorders were less common in participants who responded to the follow-up assessment (follow-up group) than in those who did not (dropout group). However, all the other characteristics were comparable. In the follow-up group, both CERAD total score and MMSE scores decreased and the frequency of cognitive disorders increased during the follow-up period. At the baseline assessment, the follow-up group showed slightly thicker retinal layers than the dropout group in the macular RNFL inner layer (mean [SD], 94.7 [14.0] vs 90.4 [12.4]; *P* < .001), all layers of the macular RNFL mean ([SD], 257.2 [34.7] vs 250.2 [34.9]; *P* = .04), ganglion cell outer layer mean ([SD], 131.3 [18.3] vs 135.6 [19.8]; *P* = .02), and inner nuclear inner layer mean ([SD], 160.1 [15.6] vs 156.9 [15.7]; *P* = .04) (eTable 1 in the Supplement).

**Table 1.** Demographic and Clinical Characteristics of the Participants

Characteristic	All (N = 430)	Dropout group (n = 215)	No. (%), follow-up group (n = 215)	
			Baseline	Follow-up
Age, mean (SD), y	76.3 (6.6)	76.2 (6.5)	76.5 (6.7)	80.9 (6.5)
Sex, No. (%)				
Women	208 (48.6)	102 (47.9)	106 (49.3)	NA
Men	222 (51.4)	113 (52.1)	50.7 (109)	NA
Education, mean (SD), y	9.1 (5.6)	9.2 (5.9)	9.4 (5.7)	NA
Axial length, mean (SD), mm	23.4 (1.2)	23.5 (1.4)	23.1 (1.3)	NA
IOP, mean (SD), mm Hg	12.1 (3.3)	11.9 (3.5)	12.6 (3.1)	NA
*APOE ε4*, No. (%)	70 (16.2)	30 (14.0)	40 (22.8)	NA
Diabetes, No. (%)	90 (20.1)	48 (22.3)	42 (19.5)	NA
Hypertension, No. (%)	170 (29.5)	80 (37.2)	90 (41.9)	NA
CERAD total score, mean (SD)	66.7 (12.1)	66.0 (16.5)	66.6 (11.2)	54.1 (11.4)
MMSE score, mean (SD)	24.5 (4.6)	24.4 (5.1)	25.2 (3.2)	21.5 (3.1)
Cognitive disorders, No. (%)				
MCI	63 (14.7)	38 (17.7)	25 (11.6)	38 (17.7)
Alzheimer disease	12 (2.8)	6 (2.8)	6 (2.8)	9 (4.2)
All	75 (17.5)	44 (20.4)	31 (14.4)	47 (21.9)

Abbreviations: *APOE*, apolipoprotein E; CERAD, Consortium to Establish a Registry for Alzheimer’s Disease Neuropsychological Battery; IOP, intraocular pressure; MCI, mild cognitive impairment; MMSE, Mini-Mental State Examination.

At baseline, the macular RNFL thickness was associated with the baseline CERAD total score (baseline CERAD total score for outer thickness: β = 0.071; 95% CI, 0.047-0.095; *P* = .04; baseline CERAD total score for inner thickness, β = 0.069; 95% CI, 0.051-0.087; *P* = .047; baseline CERAD total score for total thickness: β = 0.077; 95% CI, 0.054-0.100; *P* = .04) and the baseline MMSE score (MMSE score for outer thickness: β = 0.078; 95% CI, 0.059-0.09; *P* = .04; MMSE score for inner thickness: β = 0.074; 95% CI, 0.058-0.090; *P* = .04; MMSE score for total thickness: β = 0.082; 95% CI, 0.063-0.101; *P* = .03) (Table 2). In the longitudinal study, macular RNFL thickness was not associated with decreases in the CERAD total score and MMSE scores during the follow-up period: outer RNFL CERAD total score: *F*_208_ = 1.170; 95% CI, 0.845-1.495; *P* = .21; MMSE: *F*_208_ = 0.956; 95% CI, 0.637-1.275; *P* = .59; inner RNFL CERAD total score: *F*_208_ = 1.229; 95% CI, 0.896-1.52; *P* = .16; MMSE: *F*_208_ = 1.358; 95% CI, 0.958-1.758; *P* = .01; total RNFL CERAD total score: *F*_208_ = 1.420; 95% CI, 0.971-1.869; *P* = .07; and MMSE: *F*_208_ = 0.879; 95% CI, 0.582-1.176; *P* = .74 (Table 3).

**Table 2.** Association of Baseline Retinal Layer Thickness With Baseline Cognitive Performance^a^

Characteristic	CERAD total score		MMSE score	
	β	*P* value	β	*P* value
Subfoveal choroid	0.021	.40	0.024	.42
Retinal nerve fiber layer				
Average peripapillary	0.034	.29	0.015	.56
Macular				
Outer	0.071	.04	0.078	.04
Inner	0.069	.047	0.074	.04
Total	0.077	.04	0.082	.03
Ganglion cell layer				
Outer	0.004	.91	0.016	.65
Inner	0.001	.98	0.018	.62
Total	0.003	.94	0.004	.92
Inner plexiform layer				
Outer	0.008	.82	0.006	.87
Inner	0.006	.87	0.049	.19
Total	0.008	.82	0.026	.47
Inner nuclear layer				
Outer	0.012	.72	0.039	.29
Inner	0.032	.36	0.021	.57
Total	0.015	.67	0.033	.37
Outer plexiform layer				
Outer	0.033	.34	0.025	.50
Inner	0.039	.26	0.022	.55
Total	0.042	.23	0.025	.50
Outer nuclear layer				
Outer	0.032	.37	0.028	.45
Inner	0.025	.48	0.052	.15
Total	0.029	.41	0.044	.23

Abbreviations: CERAD, Consortium to Establish a Registry for Alzheimer’s Disease Neuropsychological Battery; MMSE, Mini-Mental State Examination.

^a^ Multiple linear regression analyses adjusted for age, sex, level of education, diabetes, hypertension, and the presence of the apolipoprotein ε4 allele.

**Table 3.** Association of Baseline Macular Retinal Nerve Fiber Layer Thickness With Changes in Cognitive Performance During the Follow-up Period^a^

Characteristic	CERAD total score		MMSE	
	*F*_208_	*P* value	*F*_208_	*P* value
Outer				
Thickness	1.170	.21	0.956	.56
Lowest quartile (135 μm)	6.912	.01	4.265	.04
Inner				
Thickness	1.229	.16	1.358	.09
Lowest quartile (86 μm)	7.600	.006	6.626	.01
Total				
Thickness	1.420	.07	0.879	.74
Lowest quartile (231 μm)	8.980	.003	6.645	.01

Abbreviations: CERAD, Consortium to Establish a Registry for Alzheimer’s Disease Neuropsychological Battery; MMSE, Mini-Mental State Examination.

^a^ Repeated-measures analysis of variance.

**Figure 1.**
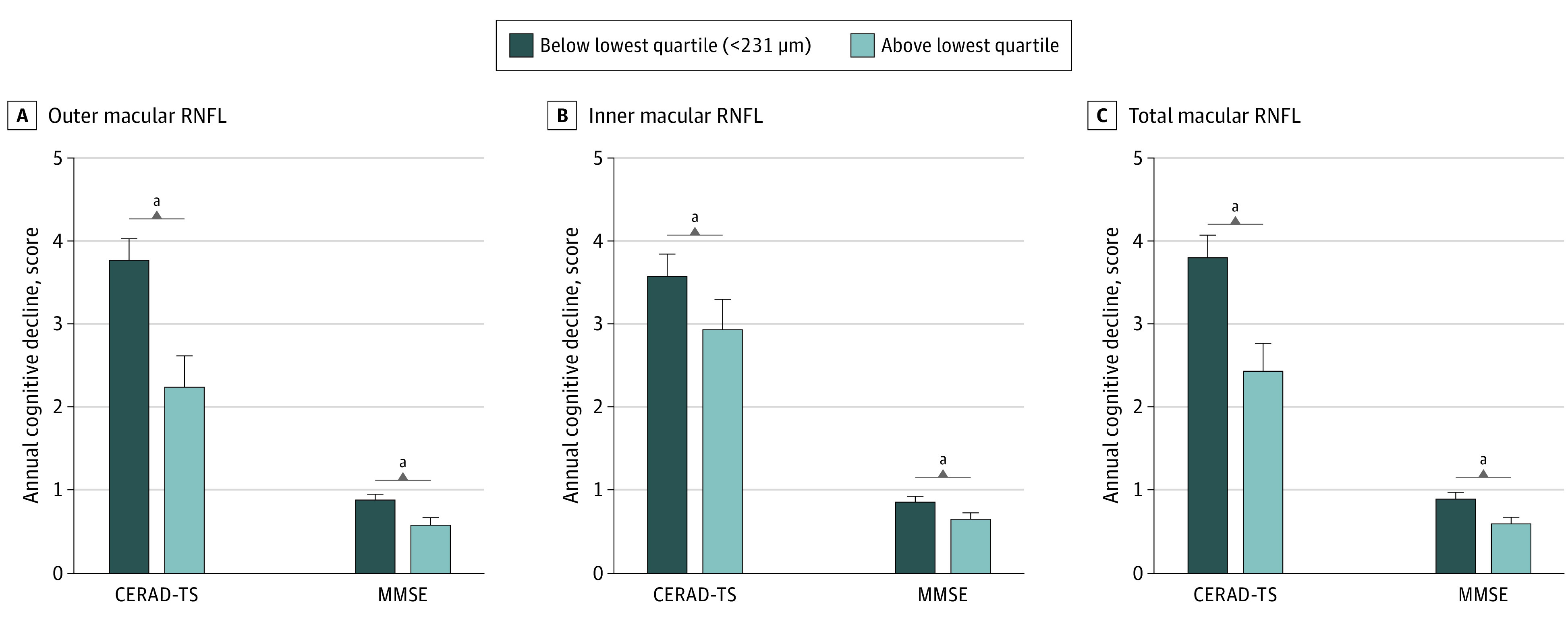
Decline in Annual Cognitive Performance Scores According to the Baseline Total Macular Retinal Nerve Fiber Layer (RNFL) Quartile Groups Outer, inner, and total macular RNFL thickness data were subdivided into 2 groups, ie, below the lowest quartile and above the lowest quartile. The degree of cognitive function decline was higher in the below-lowest-quartile group (thinner macular RNFL). Abbreviations: CERAD-TS indicates Consortium to Establish a Registry for Alzheimer’s Disease Neuropsychological Battery total score; MMSE, Mini-Mental State Examination. ^a^P < .05.

**Figure 2.**
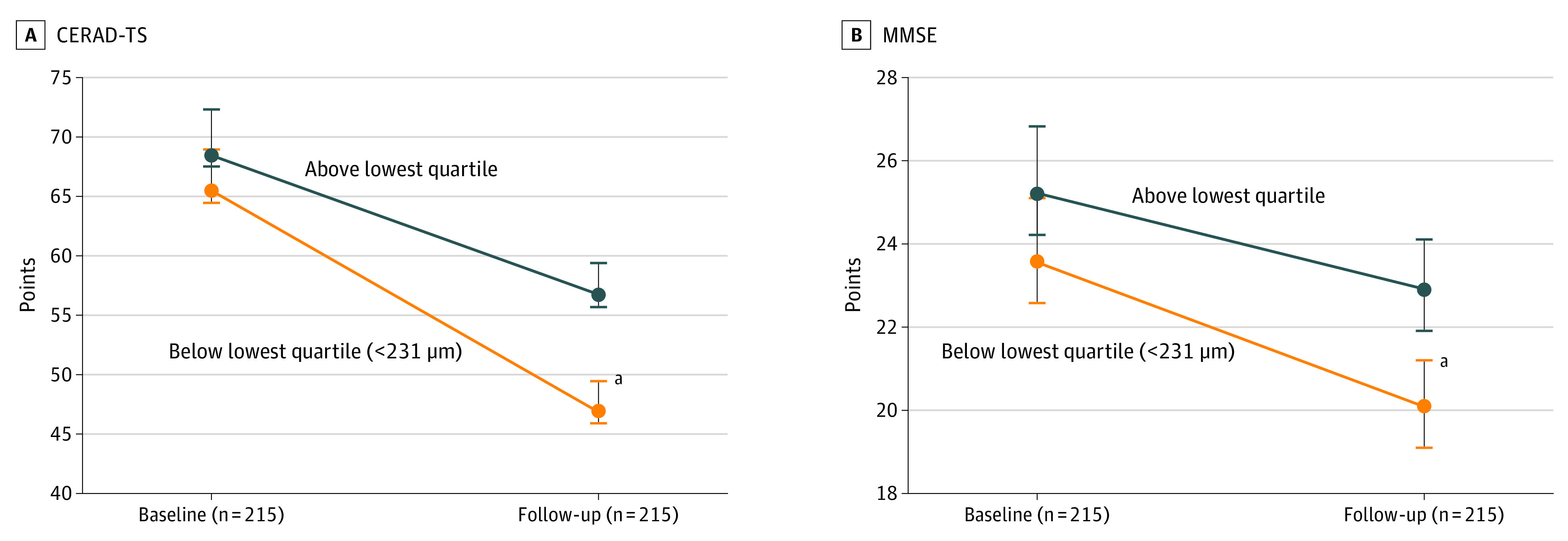
Cognitive Performance Scores According to the Baseline Total Macular Retinal Nerve Fiber Layer (RNFL) Quartile Groups Both Consortium to Establish a Registry for Alzheimer’s Disease Neuropsychological Battery total score and Mini-Mental State Examination scores were not different at baseline; however, they differed at the follow-up evaluation. The below-lowest-quartile group showed a greater cognitive decline than the above-lowest-quartile group. ^a^*P *< .05.

However, after setting the cutoff value as the lowest quartile (outer, 135 μm; inner, 86 μm; total, 231 μm), the baseline macular RNFL thickness was associated with a decrease in both CERAD total score and MMSE scores during the follow-up period. A thinner baseline outer RNFL was associated with a decline in both CERAD total score (*F*_208_ = 6.912; 95% CI, 5.478-8.346; *P* = .009) and MMSE scores (*F*_208_ = 4.265; 95% CI, 3.189-5.341; *P* = .04); the inner RNFL in both CERAD total score (*F*_208_ = 7.600; 95% CI, 5.913-9.287; *P* = .006); MMSE scores (*F*_208_ = 6.626; 95% CI, 5.237-8.015; *P* = .01); and the total RNFL in both CERAD total score (*F*_208_ = 8.980; 95% CI, 6.792-11.168; *P* = .003); and MMSE scores (*F*_208_ = 6.645; 95% CI, 4.968-8.322; *P* = .01) (Table 3). The decline in annual cognitive performance scores according to baseline macular RNFL is shown in Figure 1 and eTable 2 in the Supplement. In addition, the baseline and follow-up CERAD total scores and MMSE scores divided by the lowest quartile of total macular RNFL (<231 μm) are shown in Figure 2 and eTable 2 in the Supplement. Interestingly, the cognitive performance scores were not different at baseline but showed differences at follow-up. Figures 1 and 2 show that participants with a thinner baseline macular RNFL were likely to experience a greater degree of cognitive decline, leading to cognitive impairment.

The baseline and follow-up prevalence of MCI and AD according to the lowest quartile are shown in eTable 2 in the Supplement. In the lower-quartile group (n = 55), the prevalence of MCI increased from baseline from 27.2% (15 of 55) to 41.8% (23 of 55) and the prevalence of AD increased from 7.2% (4 of 55) to 10.9% (6 of 55) at follow-up, whereas in the above-lowest-quartile group (n = 160), the prevalence of MCI increased from 6.3% (10 of 160) to 9.4% (15 of 160) for MCI and from 1.3% (2 of 160) to 1.9% (3 of 160) for AD at follow-up. The below-lowest-quartile group showed a much higher rate of MCI and AD prevalence than the above-lowest-quartile group.

## Discussion

This longitudinal cohort study enrolled older community-based participants to attempt to reveal the association between OCT-measured retinal layer thickness and cognitive function and decline. The results of the cross-sectional study showed that the change in thicknesses of the outer and inner macular RNFLs were associated with the baseline CERAD total scores and MMSE scores (Table 2). In the longitudinal study, the overall baseline macular RNFL thickness was not associated with baseline cognitive performance scores. Instead, after setting the cutoff value as the lowest quartile, the baseline outer, inner, and total macular RNFL thicknesses were associated with changes in both CERAD total scores and MMSE scores (Table 3). Moreover, there were no differences in the cognitive score and RNFL thickness according to *APOE* ε4 status (eTable 3 in the Supplement).

Previously, researchers found that retinal layer thickness in the RNFL, GC-IPL, and other areas was related to cognitive impairment; thus, retinal layer assessment with OCT could be a convenient method of cognitive function assesment.^8,9,10,12,17,20^ However, recent studies on patients with preclinical AD have reported some conflicting results. Snyder et al^31^ observed a thick IPL in patients with preclinical AD, whereas López-Cuenca et al^32^ observed a thin macular RNFL, IPL, inner nuclear layer, and outer plexiform layer. In contrast, van de Kreeke et al^33,34^ showed no differences in any of the measured retinal layers, including the peripapillary and macular RNFL, ganglion cell layer, and IPL. Therefore, an exploratory cross-sectional analysis was performed to discover which retinal layer is relevant for cognitive performance. We found that only the macular RFNL was associated with neuropsychiatric test scores and that it could be a candidate layer for predicting cognitive decline.

Unlike previous investigations focusing on patients with MCI and AD, these longitudinal population-based studies included older participants from the general population in local communities to detect possible associations between retinal layer thickness and early changes in cognitive function. Méndez-Gómez et al^35^ from the Three-City-Alienor cohort (427 participants) proposed that RNFL thickness was not associated with initial cognitive scores using the MMSE, but it was associated with episodic memory loss over 2 years of follow-up. A UK Biobank prospective, multicenter, community-based study^21^ reported that a thinner RNFL was associated with worse cognitive function at baseline and during follow-up and presented an increased risk of dementia. The UK Biobank study examined large cohorts of more than 30 000 individuals at baseline and 1251 individuals at the 3-year follow-up using cognitive tests including prospective memory, pairs matching, numeric and verbal reasoning, and reaction time with touch screens. The Rotterdam study^36^ analyzed more than 3000 participants and revealed that a thinner GC-IPL was associated with dementia, whereas a thinner RNFL at baseline was associated with an increased risk of developing dementia. Along with these population-based longitudinal cohort studies, we assessed the cognitive performance of the enrolled participants at baseline and final follow-up at 4 to 6 years and matched it with the baseline retinal layer thickness data.

Recent OCT angiography studies that investigated the association between OCT angiography parameters and cognitive decline showed that the FAZ area is enlarged in patients who had preclinical AD with positive biomarkers.^22,23,24^ Our study is in line with the OCT angiography studies as macular microvasculature changes, including in FAZ areas, are known to be correlated with macular RNFL thickness.^37^ However, it is still unknown whether enlargement of the FAZ precedes macular RNFL thinning, which is the main mechanism of the association between cognitive decline and macular retinal changes in the microvasculature and neural structures.

The participants of the current study were community-based people 60 years and older; thus, unlike in prior investigations, most participants presented with normal cognitive function or MCI, with only 12 patients (2.8%) having definite AD. Therefore, we considered our cohort population suitable for assessing the association between retinal layer thickness and cognitive function in a real-world clinical setting. Our results showed that baseline macular RNFL thickness was associated with baseline neuropsychiatric test scores. However, contrary to other studies, the average peripapillary RNFL thickness showed no differences. We presumed that macular RNFL thinning might precede peripapillary RNFL thinning in the early phase of cortical degeneration.

Another characteristic of our longitudinal study was that we enrolled a larger number of participants than previous case-control studies. Moreover, because we adjusted for parameters including age, sex, education level, diabetes, hypertension, and *APOE ε4 *status—that is, variables that might affect cognitive function assessment—we conducted more comprehensive statistical analyses compared with prior investigations. In addition, we investigated the possibility of predicting cognitive function decline based on baseline retinal layer thickness in a longitudinal study and found that baseline macular RNFL thickness may be associated with the prognosis of cognitive function.

Another strength of our study was that we analyzed both cognitive function assessment scores (CERAD and MMSE). Although the MMSE has certain disadvantages, such as limited examination of visuospatial cognitive ability, bias against poorly educated participants, and poor sensitivity in detecting patients with very mild dementia, it is a quick and convenient test to diagnose cognitive impairment in a clinical setting. Hence, our results could be useful for clinicians, both neuropsychiatrists and ophthalmologists, in matching OCT retinal thickness data and MMSE scores in the real world.

### Limitations

This study has limitations. First, this longitudinal study did not perform β-amyloid (Aβ) positron emission tomography and magnetic resonance imaging , as cerebral Aβ deposition starts approximately 15 to 20 years before the onset of AD, which is crucial for detecting future candidates for AD who are still cognitively healthy.^38^ van de Kreeke et al^33,34^ studied cognitively normal individuals with amyloid pathology on positron emission tomography (preclinical AD) and observed no differences in retinal layer thickness in the macular and peripapillary RNFL in both cross-sectional and longitudinal studies. In contrast, Byun et al^39^ recently reported that Aβ-positive patients with preclinical AD showed reduced inner nasal macular thickness and RNFL thickness compared with Aβ-negative patients.^39^ Second, numerous parameters besides the selected confounding variables of age, sex, education level, diabetes, hypertension, and *APOE ε4* status could also influence cognitive function; thus, strict adjustments might not be feasible. Third, in the follow-up period, baseline macular RNFL thickness as a score changes variable was not associated with either CERAD or MMSE; instead, setting the lowest quartile as the cutoff value revealed a correlation between these 2 parameters. Further longitudinal studies with larger sample sizes are warranted. Fourth, among the baseline 430 participants, only half participated in the follow-up study. This may have affected the longitudinal analysis in our cohort study. Fifth, our longitudinal study follow-up lasted for a mean (SD) of 5.4 (0.6) years (range, 4.1-6.2 years), which is a considerably small period of time in the development of neurodegenerative diseases. Subsequent follow-up at an additional time point of 5 years, approximately 10 years from baseline, was planned in this cohort study. Additionally, we could not perform the final follow-up SD-OCT evaluation; therefore, in the longitudinal study, we could not correlate changes in cognitive function score with changes in retinal layer thickness. Further investigations are needed to evaluate OCT retinal thickness changes throughout regular follow-up intervals.

### Conclusion

In conclusion, this longitudinal cohort study revealed that OCT-measured baseline macular RNFL thickness was associated with future cognitive decline. We propose that a thinner macular RNFL may predict a decline in cognitive performance. Overall, macular RNFL thickness may be considered a noninvasive ocular biomarker for assessing changes in cognitive function in patients. However, regarding the limitations mentioned above, further population-based investigations with a long-term follow-up are necessary.

## Notes

Supplement. eMethod 1. Population-based cohorts, KLOSHA and KLOSCAD. eMethod 2. Cognitive performance scores, CERAD-K and MMSE. eTable 1. Baseline retinal layer thickness data of the participants. eTable 2. Differences in cognitive performance between the below-lowest-quartile and the above-lowest-quartile groups. eTable 3. Baseline and follow-up cognitive scores and RNFL thickness according to the ApoE4 status. eFigure 1. Flow chart of patient enrollment in the KLOSHA/KLOSCAD longitudinal cohort study. eFigure 2. ETDRS fields and OCT retinal layer analysis.
