# Supplementary material for: Association Between Retinal Layer Thickness and Cognitive Decline in Older Adults
Source: JAMA Ophthalmol. 2022 May 26;140(7):683–90. doi: 10.1001/jamaophthalmol.2022.1563 (PMC9136677; doi:10.1001/jamaophthalmol.2022.1563)
Supplement: Supplement. — eMethod 1. Population-based cohorts, KLOSHA and KLOSCAD. eMethod 2. Cognitive performance scores, CERAD-K and MMSE. eTable 1. Baseline retinal layer thickness data of the participants. eTable 2. Differences in cognitive performance between the below-lowest-quartile and the above-lowest-quartile groups. eTable 3. Baseline and follow-up cognitive scores and RNFL thickness according to the ApoE4 status. eFigure 1. Flow chart of patient enrollment in the KLOSHA/KLOSCAD longitudinal cohort study. eFigure 2. ETDRS fields and OCT retinal layer analysis. [file jamaophthalmol-e221563-s001.pdf]

# Supplemental Online Content

Kim HM, Han JW, Park YJ, et al. Association between retinal layer thickness and cognitive decline in older adults. *JAMA Ophthalmol*. Published online May 26, 2022. doi:10.1001/jamaophthalmol.2022.1563

**eMethods 1.** Population-based cohorts, KLOSHA and KLOSCAD.

**eMethods 2.** Cognitive performance scores, CERAD-K and MMSE.

**eTable 1.** Baseline retinal layer thickness data of the participants.

**eTable 2.** Differences in cognitive performance between the below-lowest-quartile and the above-lowest-quartile groups.

**eTable 3.** Baseline and follow-up cognitive scores and RNFL thickness according to the ApoE4 status.

**eFigure 1.** Flow chart of patient enrollment in the KLOSHA/KLOSCAD longitudinal cohort study.

**eFigure 2.** ETDRS fields and OCT retinal layer analysis.

This supplemental material has been provided by the authors to give readers additional information about their work.

© 2022 Kim et al. *JAMA Ophthalmology.*

Supplementary Method 1 Population-based cohorts, KLOSHA and KLOSCAD.

In the KLoSHA, 714 community-dwelling Koreans aged 65 years were randomly selected from the residents of Seongnam, South Korea, in 2005, and were first followed up in 2010 and every 2 years thereafter until 2020 at SNUBH. In the KLOSCAD, 6,818 community-dwelling Koreans aged 60 years were randomly selected from the residents of 13 districts across South Korea in 2010 and followed up every 2 years at 12 university hospitals, including SNUBH, until 2020. The current SNUBH Eye Study included 500 participants who agreed to receive ophthalmic evaluations, specifically 180 participants from the KLOSHA and 320 from the KLOSCAD who were living in the city of Seongnam and Yongin.

© 2022 Kim et al. *JAMA Ophthalmology.*

Supplementary Method 2 Cognitive performance scores, CERAD-K and MMSE.

Neuropsychological Assessment Battery (CERAD-K-N), Digit Span Test, and Frontal Assessment Battery. The CERAD-K-N consists of the Verbal Fluency Test (VFT), 15-item Boston Naming Test (BNT), Mini-Mental Status Examination (MMSE), Word List Memory Test (WLMT), Constructional Praxis Test (CPT), Word List Recall Test (WLRT), Word List Recognition Test (WLRcT), Constructional Recall Test, and Trail Making Test A/B. We calculated the CERAD-K total score (CERAD-TS) by summing the scores of the VFT, BNT, WLMT, WLRT, WLRcT, CPT and the CERAD memory score (CERAD-MS) by summing the WLMT/3, WLRT, and WLRcT scores. Geriatric neuropsychiatrists with expertise in dementia research performed face-to-face standardized diagnostic interviews as well as physical and neurological examinations using the CERAD-K Clinical Assessment Battery to diagnose cognitive disorders.

© 2022 Kim et al. *JAMA Ophthalmology.*

Supplementary Table 1. Baseline retinal layer thickness data of the participants.

| | Baseline (N = 430) | Drop-out group (N = 215)[a] | Follow-up group (N = 215)[b] |
|---|---|---|---|
| Subfoveal choroid | 180.2±67.1 | 184.0±69.2 | 176.6±64.7 |
| Retinal nerve fiber layer | | | |
| Average peripapillary | 96.7±11.4 | 97.6±12.6 | 95.8±10.1 |
| Macular | | | |
| Outer | 151.5±24.7 | 149.7±24.9 | 152.4±23.4 |
| Inner | 92.7±13.6 | 90.4±12.4 | 94.7±14.0 |
| Total | 253.6±34.9 | 250.2±34.9 | 257.2±34.7 |
| Ganglion cell layer | | | |
| Outer | 133.5±19.2 | 135.6±19.8 | 131.3±18.3 |
| Inner | 186.5±24.9 | 185.0±24.1 | 187.8±25.6 |
| Total | 334.9±37.1 | 335.6±37.1 | 334.2±37.1 |
| Inner plexiform layer | | | |
| Outer | 111.4±13.5 | 112.6±14.3 | 110.1±12.6 |
| Inner | 152.9±15.2 | 152.3±15.2 | 153.6±15.2 |
| Total | 279.3±24.9 | 279.9±25.7 | 278.7±24.1 |
| Inner nuclear layer | | | |
| Outer | 127.2±11.9 | 127.7±11.9 | 126.7±11.9 |
| Inner | 158.5±15.7 | 156.9±15.7 | 160.1±15.6 |

© 2022 Kim et al. *JAMA Ophthalmology.*

| | | | |
|---|---|---|---|
| Total | 305.7±24.2 | 304.7±24.0 | 306.8±24.5 |
| Outer plexiform layer | | | |
| Outer | 109.6±9.0 | 109.1±9.2 | 109.9±8.8 |
| Inner | 137.8±20.5 | 136.1±20.4 | 139.5±20.4 |
| Total | 267.3±26.5 | 265.3±26.4 | 269.4±26.5 |
| Outer nuclear layer | | | |
| Outer | 205.7±26.8 | 206.7±28.0 | 204.7±25.6 |
| Inner | 262.6±36.1 | 263.1±38.2 | 262.0±33.8 |
| Total | 498.3±59.7 | 499.8±63.4 | 496.7±55.8 |

*Student t test for continuous variables and chi square test for categorical variables
†paired t test for continuous variables

© 2022 Kim et al. *JAMA Ophthalmology.*

Supplementary Table 2. Differences in cognitive performance between the below-lowest-quartile and the above-lowest-quartile groups

| | Below lowest quartile(<231 μm) (n = 55) | | Above lowest quartile(≥231 μm) (n = 160) | |
|---|---|---|---|---|
| Age (years) | 75.9±6.7 | | 76.5±6.5 | |
| Women (%) | 52.4 | | 47.2 | |
| Education (years) | 9.2±5.7 | | 9.1±5.6 | |
| | CERAD-TS | MMSE | CERAD-TS | MMSE |
| Annual cognitive decline Scores (Points) | 3.79±0.28 | 0.89±0.07 | 2.42±0.34 | 0.58±0.08 |
| Baseline | 65.4±3.6 | 23.6±1.5 | 68.5±3.8 | 25.2±1.6 |
| Follow-up | 46.9±2.5 | 20.1±1.1 | 56.7±2.7 | 22.9±1.2 |
| Prevalence of cognitive disorders (%) | Baseline | Follow-up | Baseline | Follow-up |
| Mild cognitive impairment | 49.1 | 69.1 | 6.3 | 9.4 |
| Alzheimer’s dementia | 10.9 | 16.4 | 1.3 | 1.9 |

CERAD-TS, Consortium to Establish a Registry for Alzheimer’s Disease Neuropsychological Battery total score; MMSE, Mini-Mental State Examination.

© 2022 Kim et al. *JAMA Ophthalmology.*

Supplementary Table 3. Baseline and follow-up cognitive scores and RNFL thickness according to the ApoE4 status

| | Baseline | | | Follow-up | | |
|---|---|---|---|---|---|---|
| | ApoE4 negative (N=360) | ApoE4 positive (N=70) | *p*-value | ApoE4 negative (N=185) | ApoE4 positive (N=30) | *p*-value |
| CERAD-TS | 66.9±15.3 | 66.9±15.3 | .661 | 54.1±14.5 | 52.9±14.4 | .780 |
| MMSE | 24.7±4.5 | 24.5±4.7 | .478 | 22.0±4.1 | 21.4±3.5 | .529 |
| RNFL thickness | | | | | | |
| Outer | 151.1±24.9 | 150.9±20.2 | .169 | 155.0±25.8 | 147.8±19.0 | .315 |
| Inner | 92.5±13.2 | 92.8±14.2 | .832 | 90.6±12.8 | 89.3±9.7 | .221 |
| Total | 249.6±35.6 | 249.7±31.4 | .552 | 252.6±37.2 | 249.2±28.1 | .370 |

Student t test for continuous variables

© 2022 Kim et al. *JAMA Ophthalmology.*

Supplementary Figure 1 (eFigure 1)

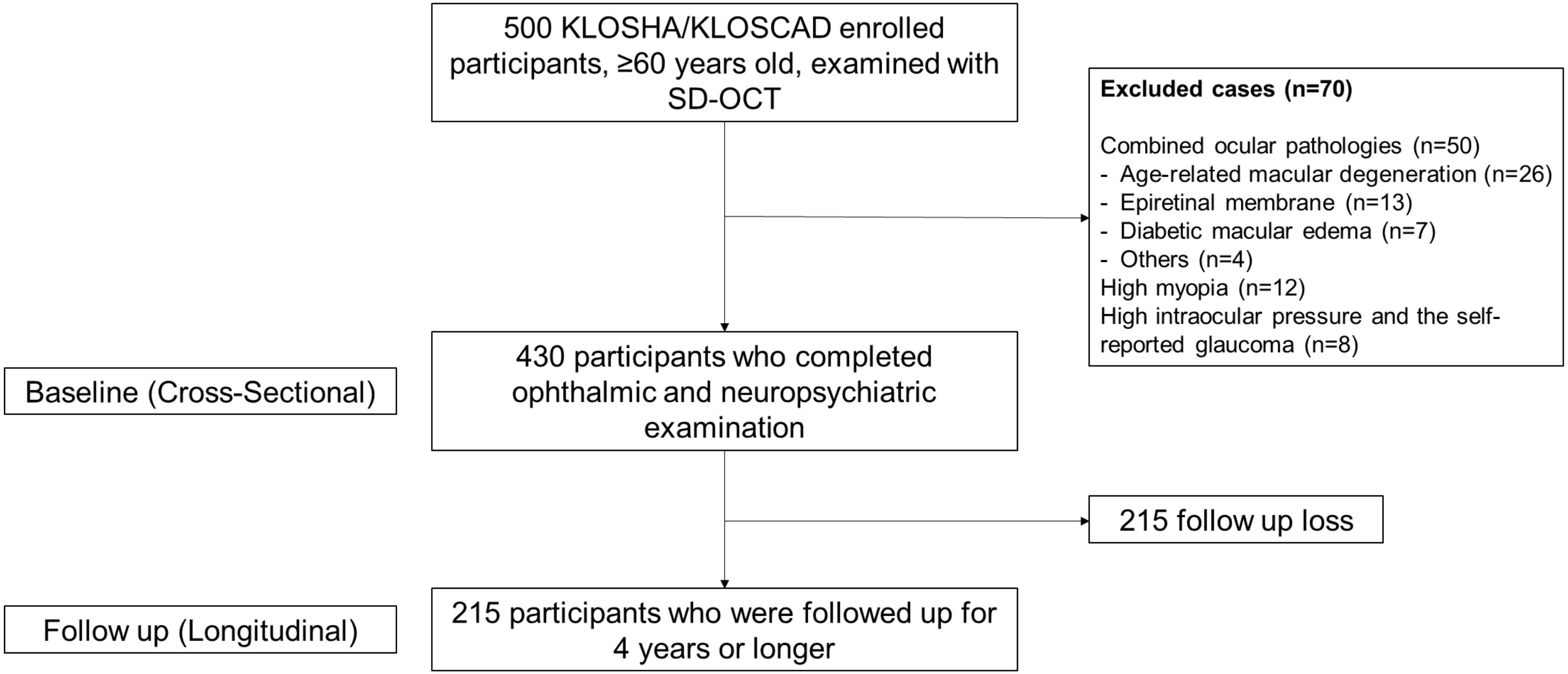


Supplementary Figure 1. Flow chart of patient enrollment in the KLOSHA/KLOSCAD longitudinal cohort study. After identifying 500 participants who underwent ophthalmic evaluation including SD-OCT, 70 participants were excluded for combined ocular pathologies (n=50), high myopia (n=12), and high intraocular pressure and self-reported glaucoma history (n=8). Consequently, the cross-sectional analysis included 430 participants. Among these, 215 who were followed up at 5- to 6-year intervals were analyzed in the longitudinal study.

© 2022 Kim et al. *JAMA Ophthalmology.*

Supplementary Figure 2 (eFigure 2)

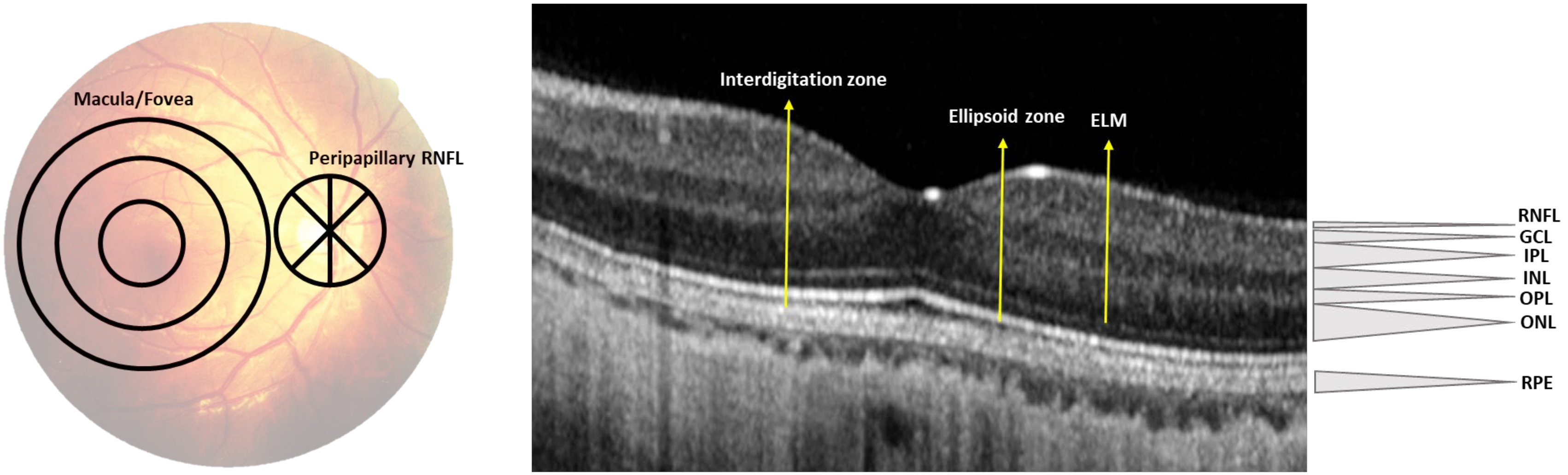


Supplementary Figure 2. (Left) Peripapillary RNFL fields and macula/fovea outer, inner, and center ETDRS fields. We measured each layer thickness and integrated them as the average peripapillary RNFL thickness and outer ring, inner ring, and total macular area macular thickness. (Right) Automated retinal layer calculation in the built-in analysis program.

© 2022 Kim et al. *JAMA Ophthalmology.*
